# On the Treatment of Neuralgia by Tobacco

**Published:** 1847-06

**Authors:** 


*On the Treatment of Neuralgia by Tobacco.*—After curing two cases of neuralgia by an infusion of tobacco, Mr. Chippendale, for convenience of application, devised an ointment which is made as follows :—Take of the best shag tobacco, four ounces ; distilled water, two pints ; boil for two or three hours, strain, and then wash the tobacco in two pints more of boiling water, strain a second time, and add it to the former liquor ; then evaporate to the consistence of an extract. One part of the extract is added to seven parts of simple cerate, to form an ointment.—*London Lancet.*

